# Slik and the Receptor Tyrosine Kinase Breathless Mediate Localized Activation of Moesin in Terminal Tracheal Cells

**DOI:** 10.1371/journal.pone.0103323

**Published:** 2014-07-25

**Authors:** Fiona Paul Ukken, Imola Aprill, N. JayaNandanan, Maria Leptin

**Affiliations:** 1 Institute of Genetics, University of Cologne, Cologne, Germany; 2 Directors' Research, European Molecular Biology Laboratory, Heidelberg, Germany; Instituto Gulbenkian de Ciência, Portugal

## Abstract

A key element in the regulation of subcellular branching and tube morphogenesis of the *Drosophila* tracheal system is the organization of the actin cytoskeleton by the ERM protein Moesin. Activation of Moesin within specific subdomains of cells, critical for its interaction with actin, is a tightly controlled process and involves regulatory inputs from membrane proteins, kinases and phosphatases. The kinases that activate Moesin in tracheal cells are not known. Here we show that the Sterile-20 like kinase Slik, enriched at the luminal membrane, is necessary for the activation of Moesin at the luminal membrane and regulates branching and subcellular tube morphogenesis of terminal cells. Our results reveal the FGF-receptor Breathless as an additional necessary cue for the activation of Moesin in terminal cells. Breathless-mediated activation of Moesin is independent of the canonical MAP kinase pathway.

## Introduction

The proteins Ezrin, Radixin and Moesin (ERM proteins) play crucial roles in cell polarity, membrane trafficking, cell-cell adhesion, cell motility, cell growth and cell shape through their interactions with the plasma membrane and the cortical actin cytoskeleton [1]–[6]. While most of the functions of ERM proteins are attributed to their ability to organize actin, ERM proteins can also regulate signaling pathways independent of their role in cytoskeleton organization [7]–[9]. ERM proteins are characterized by the presence of an N-terminal FERM domain, a central α-helical domain and a C-terminal ERM-association domain (C-ERMAD) that has the ability to bind to F-actin or form an intramolecular interaction with the N-terminal FERM domain [10]–[13]. ERM proteins exist in a closed conformation (dormant state) and the release of the C-ERMAD from the N-terminal FERM domain is necessary for their activation and interaction with F-actin [3], [10], [14]–[16]. A two-step model for ERM protein activation proposes ERM protein recruitment to the plasma membrane and phosphorylation of a conserved threonine amino acid residue in C-ERMAD as critical steps leading to their subcellular localization and activation [13], [17], [18].

The functions of ERM proteins are regulated by a battery of molecules that include factors required for their localization at the plasma membrane, intramolecular interaction between the N- and C-terminal domains of ERM and kinases and phosphatases modulating these intramolecular interactions [13], [19]. A number of membrane-localized ERM binding molecules guide ERM localization to specific subdomains within cells. The N-terminal FERM domain can bind directly to the phosphoinositide PtdIns(4,5)P2 [17], [20]–[22], to membrane proteins CD44 [23]–[25] and CD43 [23], [26], the intercellular adhesion molecule ICAM2 [23], [27], the ezrin binding phosphoprotein 50 (EBP50) [28]–[30], and the Na^+^/H^+^-exchanger NHE1 [31]. Additionally, in *Drosophila*, the apical polarity protein Crumbs and the Synaptotagmin-Like-Protein family member Bitesize (Btsz) have also been implicated in regulating membrane recruitment and ERM protein activation [32]–[35]. The two-step model explaining Moesin activation [13], [19] suggests that membrane recruitment of ERM proteins renders a regulatory threonine residue in C-ERMAD accessible for phosphorylation by specific kinases. Serine/threonine kinases including NCK interacting kinase (NIK) [36], Lymphocyte Oriented Kinase (LOK) [37], leucine-rich repeat protein kinase 2 (LRRK2) [38], CDK5, Protein Kinase Cα, Protein Kinase Cθ [39], Macrophage stimulating4 (Mst4), Rho-associated kinase [40], JNK [41] and Slik [42]–[45] can phosphorylate the threonine residue in C-ERMAD of ERM proteins. In addition, tyrosine residues in Ezrin that are conserved between mammalian ERMs are phosphorylated by the receptor tyrosine kinase -EGFR [46]–[48].

ERM proteins and the actin cytoskeleton are involved in various aspects of tubulogenesis, including branching and lumen formation in the *Drosophila* tracheal system [35], [49]–[51]. The tracheal system originates from epithelial placodes that invaginate and generate an interconnected network of branches through migration, cell shape changes and fusions. During larval development the terminal tracheal cells branch extensively, forming a highly ramified network of terminal branches with subcellular tubes [52]–[56]. A fibroblast growth factor signaling pathway, using the ligand Branchless (FGF) and the receptor Breathless (Btl), and operating through the canonical Ras/Raf/MEK/MAPK cascade, is used repeatedly during the different stages of tracheal development [54], [55], [57]–[59]. Several transcriptional targets of Bnl/Btl signaling have been identified in tracheal cells, including actin organizers such as Singed and Serum Response Factor [50], [55], [60]–[63].

Moesin, the sole *Drosophila* member of the ERM protein family, plays important roles in cytoskeleton organization, maintenance of polarity and morphogenesis of the eye, wing, salivary gland, gut and other tissues [9], [43], [64], [65]. Moesin functions at three distinct stages of tracheal morphogenesis [33], [35], [66], [67]: during tracheal placode invagination, lumen expansion and in the branching and subcellular tube morphogenesis in terminal cells. Crumbs, Btsz and the BTB-domain-containing nuclear protein Ribbon are necessary for the localization of activated Moesin to specific membrane subdomains of the tracheal cells [33], [35], [66]. The apical polarity protein Crumbs regulates Moesin localization to the apical part of invaginating cells of the tracheal placode, in a manner that is separable from Crumbs' role in establishing overall cell polarity [33]. The transcription factor Ribbon indirectly regulates active Moesin at the apical membrane and thereby affects tracheal tube elongation [66]. Btsz is critical for apical localization of activated Moesin in terminal cells as well as in fusion cells of the dorsal trunk. In *btsz* mutants the terminal cells in the larvae fail to develop terminal branches and the intracellular tubes and the fusion cells show defects in the adherens junctions [35]. Although a few factors regulating membrane localization of active Moesin have been described, the kinases that phosphorylate and activate Moesin in tracheal cells are yet to be identified.

In *Drosophila* only one kinase required for activation of Moesin has been described so far. *Drosophila* Slik was identified in a screen for genes regulating tissue growth [42]. Subsequent studies revealed that Slik positively regulates Moesin activation by phosphorylating the regulatory threonine residue in the C-ERMAD domain [30], [43]–[45]. The *Drosophila* homologue of the human tumor suppressor Neurofibromatosis-2 Merlin is another known substrate of Slik kinase [3], [44]. Merlin is negatively regulated by Slik and phosphorylation of Merlin at the membrane renders it inactive [44], [68]. These two substrates, Merlin and Moesin, functionally interact with each other through their competition for Slik at the plasma membrane [3], [44], [69], [70]. While Slik regulates Moesin and Merlin through its kinase function, a kinase independent genetic interaction of Slik with Raf has also been described [42].

Here we describe two regulators, Slik and Btl, of Moesin activation during tracheal development. We show that Slik localizes at the apical luminal membrane and is necessary for Moesin activation in tracheal cells, a function that is consistent with known functions of Slik in other cell types. We have also identified Btl as an additional novel regulator of Moesin. Our results suggest a canonical MAP kinase pathway-independent function of Btl in regulating Moesin during terminal cell development.

## Material and Methods

### Drosophila stocks

The following stocks were used: w[*]; P{w[+mC] = GAL4-*btl*.S}2, w[1118]; P{w[+mC] = UAS-Slik^kin^/TM6B,Tb [1], w[1118]; P{w[+mC] = UAS-Slik^kd^/TM6B,Tb[1] (David Hipfner), P{hsFLP}, w[1118]; P{neoFRT}42D P {w[+mC] = tubGal80; P{w[+mC] = btl-Gal4, *btl*-Moesin-mRFP, UAS-CD-GFP} T(2;3) CyO -TM6 (Mirka Uhlirova). y[1]; P{y[+mDint2]  = SUPor-P}*slik*[KG04837]/CyO; ry[506], w[1118]; *slik* ^1^/Cyo, P{GAL4-kr}2, P{UAS- GFP}; w[1118]; P{neoFRT}42D *slik* ^1^/CyO P{GAL4-*kr*}2, P{UAS- GFP}, P{w[+mC] = UAS-ras^N17^}TL1, w[1118], P{w[+mC] = UAS-Ras85D^N17^}TL1, w[1118] (Bloomington stock centre). RNAi transgenes from VDRC stock centre: *btl* (27106, 110277), *moe* (110654), *egfr* (107130), *merlin* (7161), *moesin* (37917, 110654), *slik* (43783), *ras* (106642), *raf* (107766), *mek* (107276), *erk* (43123, 109108).

### Immunohistology

Staged embryos and third instar larval samples were prepared and stained using standard protocols [35]. The following primary antibodies were used: guinea pig anti-Slik[43], mouse anti-SRF (1∶200, DSHB), rat anti-E-Cadherin (1∶200, DSHB), rabbit anti-Dof [71], antibody against phosphorylated moesin is rabbit anti P-ERM (1∶200, S3149 Cell Signaling), rabbit anti-Moesin (1: 1000, François Payre), rabbit anti-pMoesin (1: 1000, François Payre). Secondary antibodies: Alexa 468 and Alexa 568 and Alexa 647 (Molecular Probes) were used at a dilution of 1∶2000.

### Microscopy and image processing

Olympus FV1000 and Leica TCS SP2 microscopes were used for imaging with imaging and data analysis software packages FV10-ASW 2.0 Viewer and Leica Confocal Software LCS. Images were edited using Adobe Photoshop (Adobe Systems) and ImageJ software and assembled using Illustrator. All images are maximum intensity projections unless otherwise mentioned.

### Quantifications and statistics

Statistical analysis was performed using PRISM6 software. Total numbers of terminal cells (n) analyzed in individual experiments are provided in text and figures. Error bars indicate standard error (s.e.)

## Results

### Slik is required for terminal cell branching and tube morphogenesis

**Figure 1:**
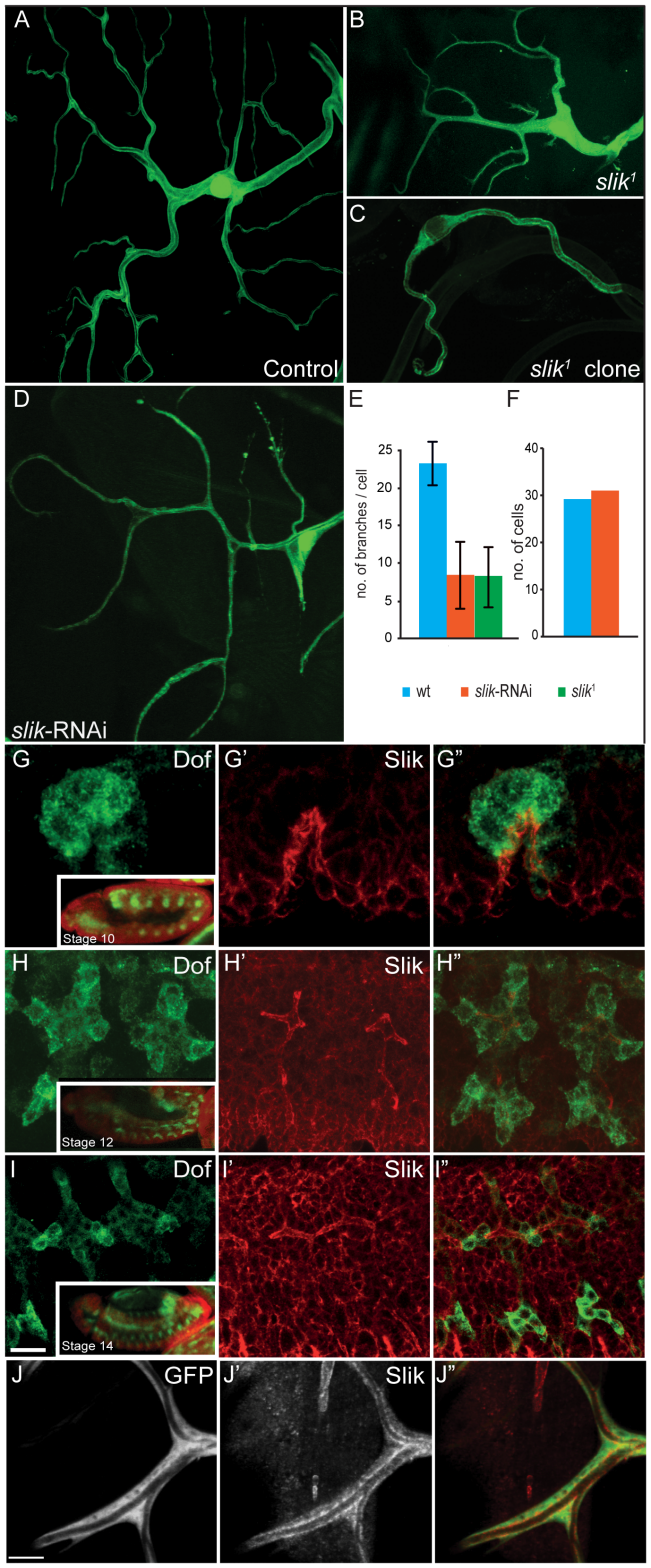
Slik is necessary for terminal cell development. (A–D) The terminal cells are labeled with cytoplasmic GFP (green). Examples of control (A), *slik* ^1^ mutant (B, C) and *slik*-RNAi (D) expressing terminal cells in third instar larvae. (E) *slik* ^1^ mutant and *slik*-RNAi expressing terminal cells have a reduced number of branches (P value <0.0001 by two tailed T test) compared to control cells. (G–J) Slik is expressed and is apically enriched in tracheal cells during different stages of embryonic development. Tracheal cells (H, I and J) are labeled by immunostaining for Dof. (J′) Slik expression in third instar larval terminal branch labeled with cytoplasmic GFP. Slik is distributed in the cytoplasm with an enrichment at the luminal membrane (J′). A–D and G–I″ are projections of confocal image stacks. (J–J″) is a single focal plane from a confocal image stack. All constructs (GFP, RNAi) are expressed under the control of the *btl*-GAL4, UAS-GFP transgene (control). Scale bars: (A–B) 50 µm, (G–I″) 10 µm, (J–J″) 5 µm.

Slik is expressed in all tracheal cells throughout development and is localized apically in embryonic tracheal cells (Fig. 1G-I″). In larval terminal cells Slik is distributed throughout the cytoplasm, with a clear enrichment at the apical luminal membrane (Fig. 1J–J″). To study the function of Slik we analyzed terminal cells in *slik* ^1^ [42] homozygous mutant larvae or MARCM clones in the tracheal system. *slik* ^1^ mutant larvae show severe general growth defects and the majority of homozygous mutants die either as larvae or pupae ([42] and Fig. S1A and B). The small number of terminal cells we were able to analyse in *slik* ^1^ homozygous mutant larvae (n  =  9) had significantly reduced numbers of branches compared to control animals (Fig. 1A, B and E). In larvae bearing *slik* ^1^ MARCM clones, mutant terminal cells were extremely rare (3 cells in 30 larvae). This is consistent with previous studies demonstrating a requirement of Slik for viability [42], [43], even though terminal cells were able to survive in *slik* ^1^ mutant larvae. It is likely that mutant terminal cells in a mosaic situation were outcompeted by their heterozygous neighbours. The few surviving cells showed almost no branching (Fig. 1C). The small size of *slik* ^1^ homozygous mutant larvae and the low number of *slik* ^1^ MARCM clones rendered analysis of terminal cells at the larval stages in these genotypes difficult. To overcome this problem we tested *slik*-RNAi transgenes using *btl-*GAL4. *slik*-RNAi resulted in a similar reduction in branching as seen in *slik* ^1^ homozygous mutants (Fig. 1 D and E). Collectively, these results indicate a cell-autonomous function of Slik in terminal cell development and show that the *slik*-RNAi transgene is a suitable tool for further analysis of Slik function in terminal cells.

### Slik is required for activation of Moesin in terminal cells

**Figure 2:**
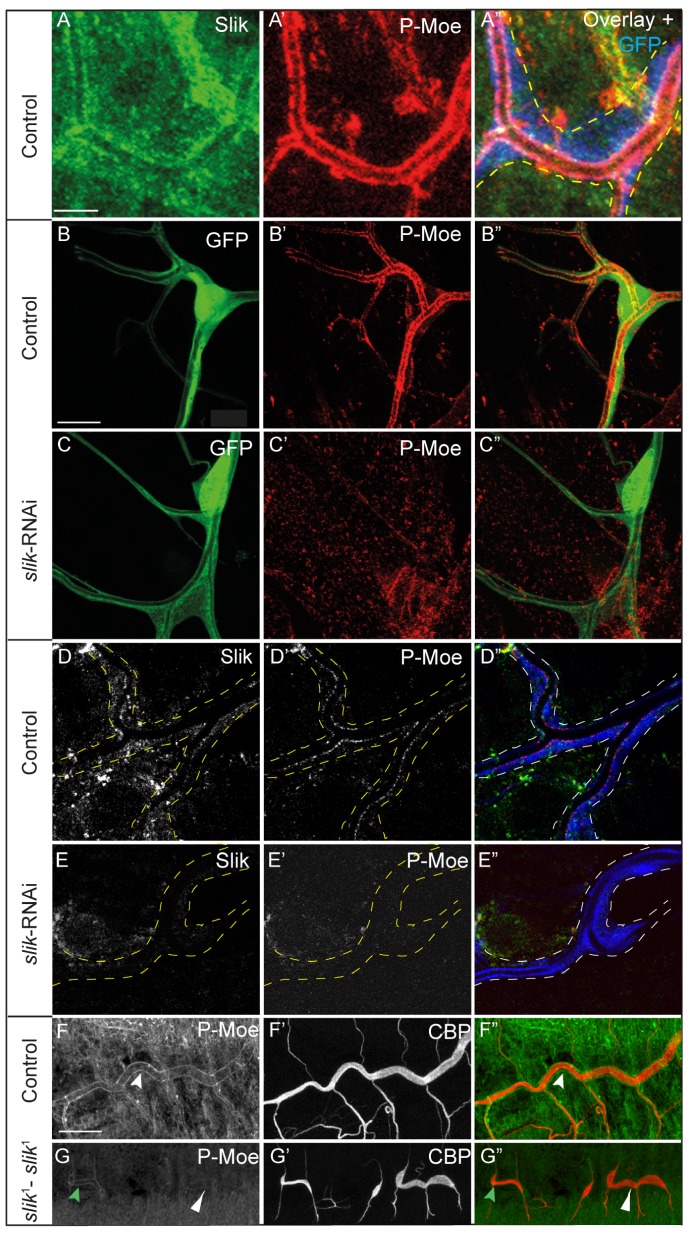
Slik activates Moesin at the luminal membrane in terminal branches. (A–A″) Region of a terminal branch from a third intar larva showing Slik (A) and activated pMoesin (A′) staining. Slik colocalizes with pMoesin at the luminal membrane (A″). The terminal cell is labeled with cytoplasmic GFP, pseudo-colored in blue (A″). (B–C″) pMoesin staining in control (B′) and Slik-depleted (C′) terminal cells. No pMoesin is seen at the luminal membrane in Slik-depleted cells (C′). (D–E″) Higher magnification of a control cell (E) and an example of a *slik*-RNAi expressing terminal cell (D) with residual Slik staining but no pMoesin. (F–G″) In *slik* ^1^ homozygous mutant embryos pMoesin staining is present in some branches (F′, green arrowhead) and reduced or absent (F′, white arrowhead) in others. The tracheal tubes are labeled with CBD-Alexa 633 (F′, F″, G and G″, pseudo-colored, in blue). The larval terminal cell is labeled with cytoplasmic GFP, pseudo-colored in blue (D″ and E″). A–C″ and F–G″ are projections of confocal image stacks. D–E″ are single focal planes from image stacks. Scale bars: (A–A″ and D–E″) 5 µm, (B–C″) 20 µm, (F–G″) 20 µm.

Localized activation of Moesin at the apical luminal membrane, mediated by Btsz, is critical for branching and tube formation during terminal cell development [35]. Moesin has been shown to be a Slik substrate in other cell types [42], [44]. We tested whether phosphorylation of Moesin may require Slik in terminal cells. Immunostaining with an antibody that recognizes threonine-phosphorylated Moesin (referred to as pMoesin here) showed that in control terminal cells pMoesin exclusively localized at the apical luminal membrane of the terminal cells where it colocalizes with Slik (Fig. 2A′ and B′). When *slik*-RNAi is expressed, most terminal cells show no Slik staining, although a small number (12%, Fig. 2E) show residual levels. In all *slik*-RNAi expressing cells pMoesin enrichment at the luminal membrane was lost (Fig. 2C′ and E′) showing that Slik is necessary for the activation of Moesin. However, Slik depletion did not lead to as severe a reduction in branching as seen in Moesin-depleted terminal cells [35]. We also analyzed pMoesin localization in embryonic tracheal branches. Apically localized pMoesin was affected in tracheal branches of *slik* ^1^ homozygous mutant embryos, but not completely disrupted in all branches (Fig. 2F and G). This variation in pMoesin is perhaps due to perdurance of maternal Slik protein in the *slik* ^1^ homozygous mutant embryos.

In addition to Moesin, another known substrate of Slik in *Drosophila* is the neurofibromatosis 2 tumor suppressor protein Merlin. We investigated whether the phenotype in Slik-depleted terminal cells was partially due to misregulation of Merlin. Merlin is widely distributed throughout the wild-type terminal cell, with local enrichments in an irregular punctate pattern (Fig. S1D′). Merlin is also expressed in the muscles, seen as a strong staining surrounding the terminal cells, which are embedded in these muscles. Neither the overall distribution nor the level of Merlin was significantly affected in Slik depleted cells (Fig. S2). We used RNAi to deplete tracheal cells of Merlin. Merlin-depleted cells had larger masses of cytoplasm around the nucleus, so that the cells appeared wider in this region than control cells (Fig. S1E). However, unlike the situation in imaginal discs [44], this phenotype was not mirrored by overexpression of Slik, which caused no tracheal defects, suggesting that Merlin does not act in the same pathway in terminal cell branching and tube formation as Slik.

### Breathless regulates Moesin in terminal cells

Despite the loss of pMoesin in *slik*-RNAi terminal cells the effect on branch development was not as severe as seen in *moe*-RNAi cells [35], suggesting the possibility of additional mechanisms regulating Moesin. The mammalian ERM-family protein Ezrin has tyrosine residues that are phosphorylated by EFGR and essential for Ezrin′s function [46]. It seemed plausible that the activation of Moesin in terminal cells could also involve other kinases in addition to Slik, perhaps tyrosine kinases. To test this hypothesis we looked at the effect of two receptor tyrosine kinases with established functions in the tracheal system, the FGF-receptor Btl and the EGF-receptor, on pMoesin levels in tracheal cells. These receptors, signaling through the canonical MAP kinase pathway, regulate several aspects of tracheal development both during embryonic and larval stages [53], [72]–[76].

**Figure 3:**
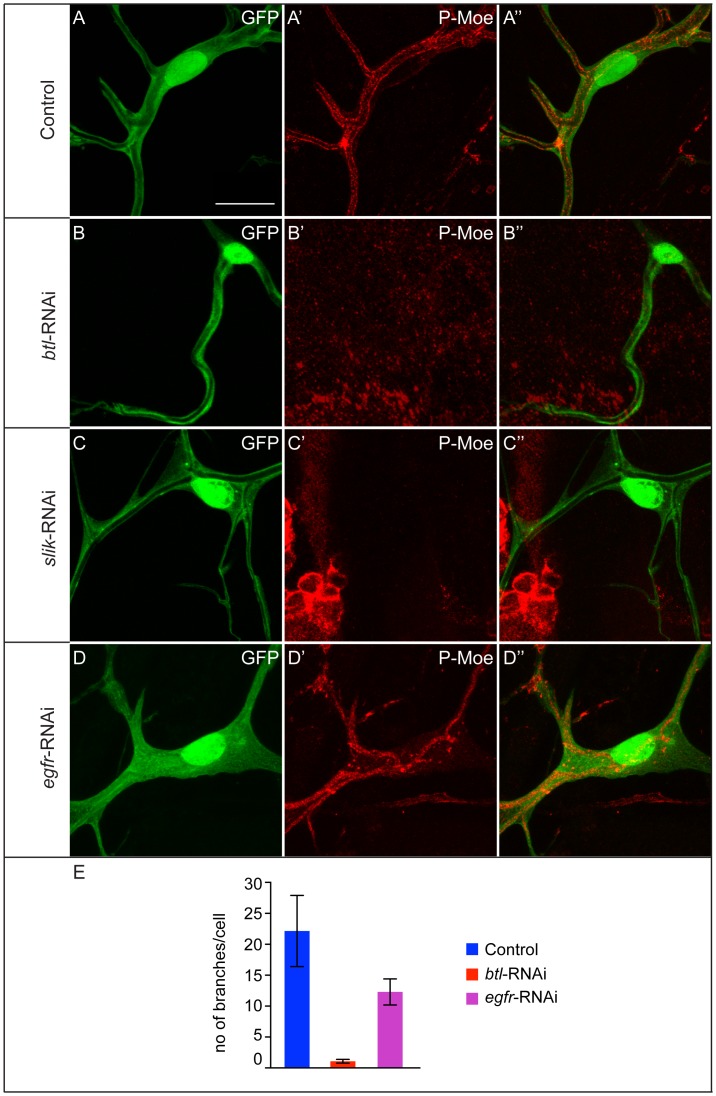
Breathless regulates pMoesin at the luminal membrane in terminal branches. (A–D″) pMoesin staining in control terminal cells (A–A″), and terminal cells expressing *btl*-RNAi (B-B″), *slik*-RNAi (C–C″) or *egfr*-RNAi (D–D″). pMoesin staining is absent in *btl*-RNAi (B′) and in *slik*-RNAi cells (C′). In *egfr*-RNAi cells (D′) pMoesin is present at levels comparable to control cells (A′). In both Btl and EGFR-depleted terminal cells branch numbers are significantly reduced (E, P value <0.0001 by two-tailed T test in both cases). In 63% of Btl-depleted cells (N = 19) pMoesin was absent, 27% showed pMoesin staining. Genotypes of crosses and number of terminal cells scored: Blue (control): *btl*-GAL4, UAS-GFP (N = 11). Red: *btl*-RNAi (N = 11). Violet: *egfr*-RNAi (n = 10). A–D″ are projections of confocal image stacks. Scale bars: (A–D″) 25 µm.

Both *btl* and *egfr* null mutants die before the third larval instar and larval cells therefore cannot be analyzed. Expression of *btl*-RNAi and *egfr*-RNAi constructs in the tracheal system affects tracheal development but allows development to proceed until pupal stages. In both cases, we observe defects in terminal cells. Most Btl-depleted cells make only one branch with a lumen (Fig. 3B and E). In a few cases the cells had long cytoplasmic extensions without any visible lumen (Fig. S3). Expression of *egfr*-RNAi constructs also resulted in a reduction in the number of terminal branches, but less extreme than observed with *btl*-RNAi (Fig. 3D and E). Many of the EGFR-depleted terminal cells had multiple lumens enclosed within individual branches, but in these cells pMoesin was present at the luminal membranes (Fig. 3D′). By contrast, in Btl-depleted cells, pMoesin was not detectable at the lumenal membrane (Fig. 3B′, F and Fig. S4). To test whether this was only one aspect of a more generally disorganized lumenal membrane, we also stained the cells for the luminal membrane protein Pio [77]. We found that Pio localization was normal (Fig. S5), suggesting that Btl depletion specifically affects the activation of Moesin. We conclude that Btl, but not the EGFR, is necessary for the activation of Moesin in terminal cells.

**Figure 4:**
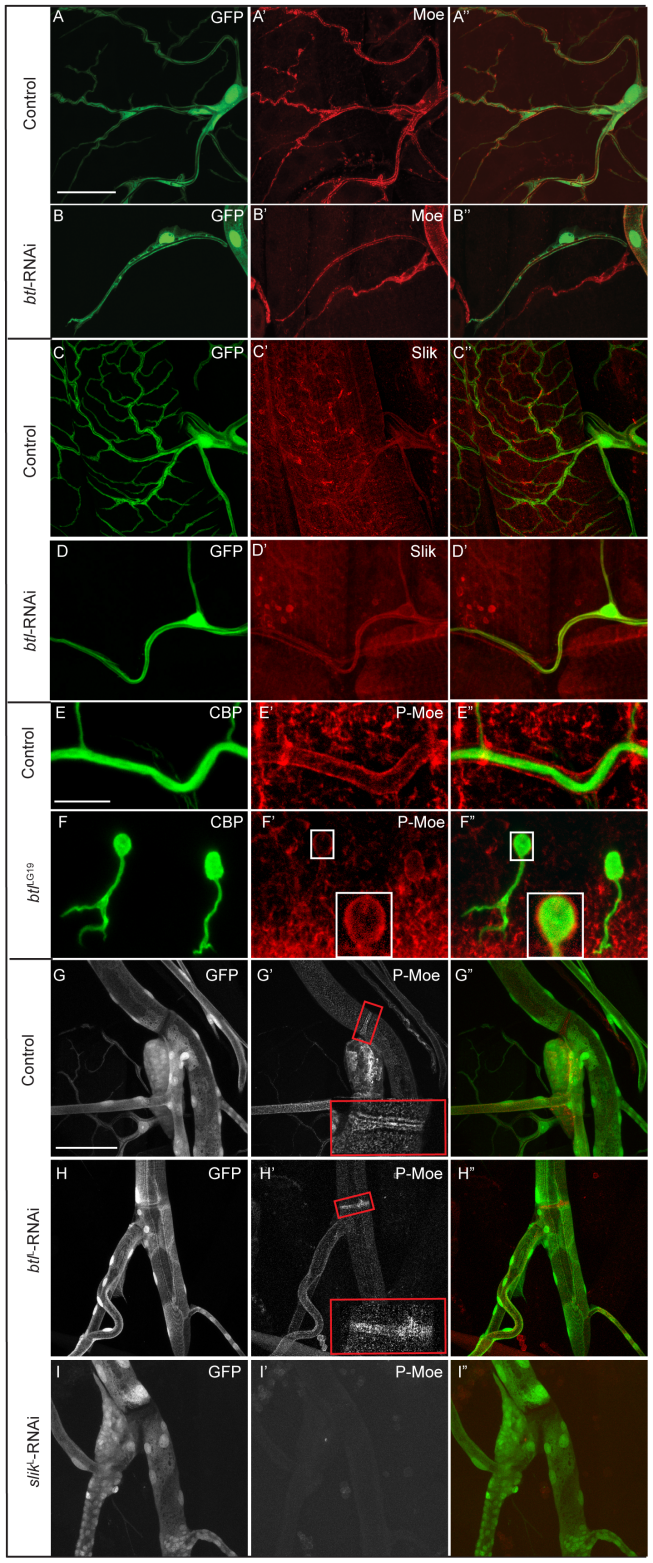
Breathless-mediated regulation of Moesin is specific for terminal cells. (A–B″) total Moesin in control (A′) and *btl*-RNAi (B′) expressing terminal cells. (C–D″) Slik staining in control (C′) and *btl*-RNAi (D′) expressing terminal cells. Depleting Btl does not affect total Moesin (B′) or Slik (D′) in terminal cells. (E–F″) pMoesin staining in control and *btl* ^LG19^ mutant embryos. pMoesin localizes apically in control (E′) and *btl* ^LG19^ (F′) mutant embryonic tracheal branches. The insets in F′ and F″ are zoomed images from the boxed regions. (G–I″) pMoesin staining in control (G), *btl-*depleted (H) and *slik*-depleted (I) larval dorsal trunk, tracheoblasts and fusion cells (boxed regions in G′ and H′). pMoesin staining in *btl-*depleted cells is comparable to the control, whereas it is absent in *slik*-depleted cells. A-I″ are projections of confocal image stacks. Scale bars: (A–D″) 50 µm, (E–I″) 25 µm.

Btl cannot be a direct kinase for the phosphorylated site on Moesin detected in the stainings since the antibody used here is specific for a phosphorylated threonine (T559) whereas Btl is a tyrosine kinase. One simple explanation for the observed effects would be that Btl signaling upregulates the levels of Slik or Moesin. We specifically tested this by immunostaining for total Moesin and Slik in Btl-depleted cells. Both Moesin and Slik are present at similar levels as in control cells (Fig. 4A-D″), showing that lack of Btl does not cause a significant overall loss of Moesin or Slik. These findings do not exclude other possible transcriptional targets of Btl-signaling regulating the observed effect on pMoesin.

We also noticed that in other parts of the tracheal system membrane-localized pMoesin was seen even in the absence of Btl signaling. In *btl* mutant embryos, pMoesin is concentrated at the inner, apical surface of the epithelial cysts that are the segmental, unfused remnants of the tracheal system (Fig. 4F′ and F″). In the dorsal trunk of *btl*-RNAi expressing third instar larvae pMoesin levels were comparable to the controls, whereas pMoesin is abolished when *slik*-RNAi is expressed in the tracheal system (Fig. 4G–I″). The requirement of Btl for the activation of Moesin at the apical plasma membrane is therefore specific for the lumenal membrane of the terminal cells.

### Epistatic relationships between Breathless, Slik and Moesin

**Figure 5:**
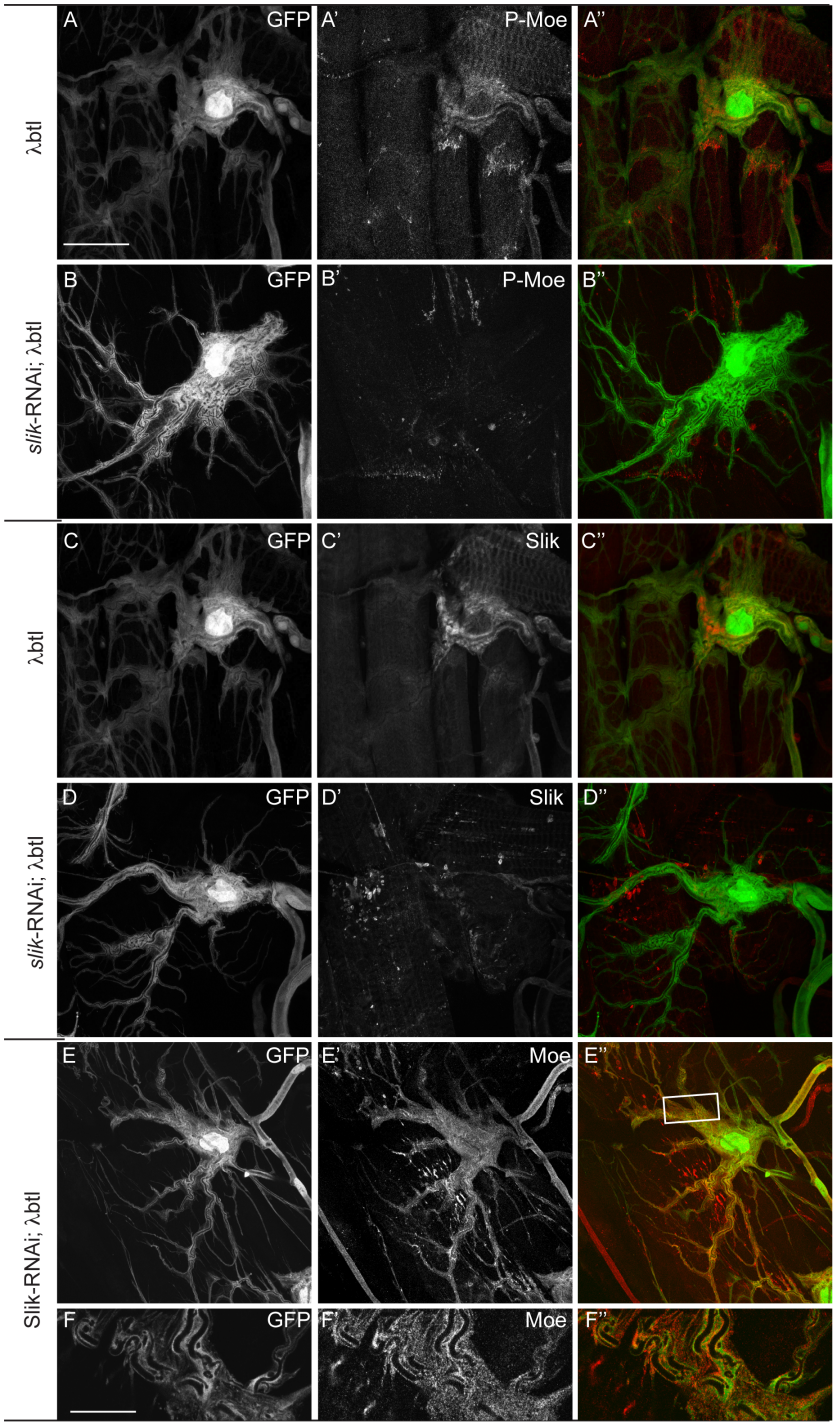
Overactivation of FGF signaling overrides requirement for Slik in terminal cell branching. (A–D) Moesin (A, B) and Slik (C–D) staining in larvae expressing constitutively active FGFR alone (λ*btl*, A′) or in combination with *slik*-RNAi (B′). λ*btl*, induces excessive branching; pMoesin and Slik staining are detectable in the λ*btl* expressing cells, (A′, C′) but absent if *slik*-RNAi is co-expressed(B′, D′). Depletion of Slik in λ*btl* expressed terminal cells does not affect expression or membrane localization of total Moesin (E and F′). A–E″ are projections of confocal image stacks. F–F″ are single focal planes from a image stack. Scale bars: (A–E″) 30 µm, (F–F″) 5 µm.

**Figure 6:**
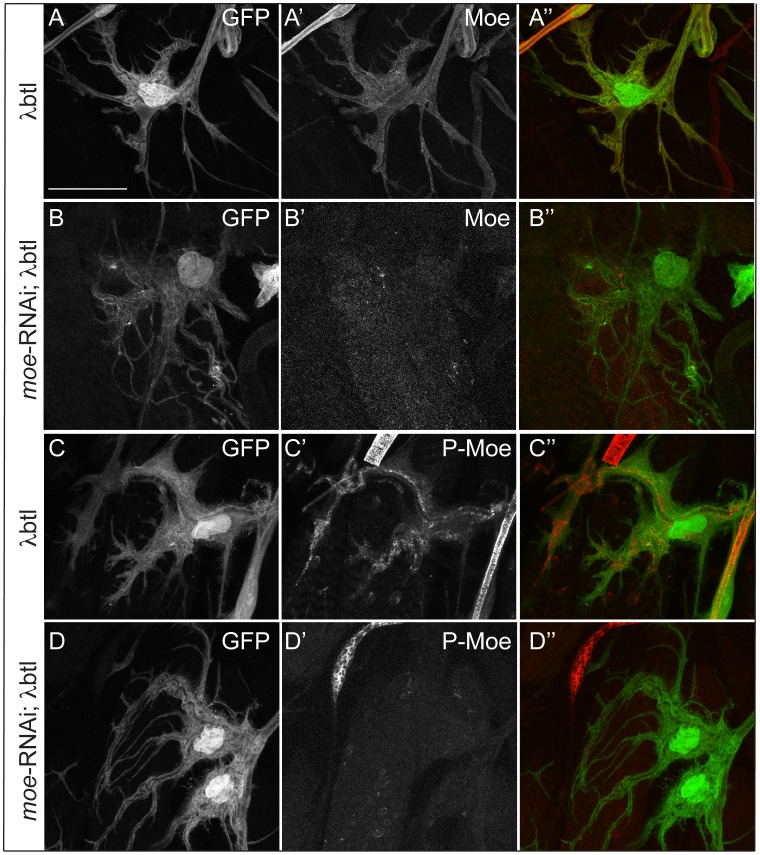
Depletion Moesin does not suppress the FGF signaling overactivation phenotype in terminal cells. (A–B″) Total Moesin staining in larvae expressing constitutively active FGFR alone (λ*btl*, A′) or in combination with *moe*-RNAi (B′) Moesin staining is detectable in the control (A′) but not in *moe*-RNAi; λ*btl* cells(B′). (C–D″) pMoesin staining in larvae expressing constitutively active FGFR alone (λ*btl*, C) or in combination with *moe*-RNAi (D′). pMoesin is absent or reduced in the *slik*-RNAi; λ*btl* cells (D′). A–D″ are projections of confocal image stacks. Scale bars: (A–D″) 40 µm.

Btl could regulate Moesin by acting in parallel to or upstream of Slik. To test if Slik acts downstream of Btl in tracheal branching, we used λBtl, a constitutively active form of Btl [78]. Expression of λBtl in terminal cells results in excessive branching and tube formation (Fig. 5A). We reasoned that if Slik is a necessary factor downstream of Btl, then depletion of Slik should modify the λBtl over-expression phenotype. However, we find that the expression of *slik*-RNAi together with λBtl did not result in any striking reduction of the excessive branching and lumen formation phenotype (Fig. 5B). It seemed possible that excessive Btl activity may have promoted the activity of residual Slik in *slik*-RNAI cells, but this is unlikely since we did not detect any residual pMoesin (Fig. 5B′), despite the normal luminal membrane localization of total Moesin (Fig. 5E′ and F′). This result therefore seems to suggest that Btl does not act through Slik to induce excessive branching. However, it was puzzling that although pMoesin was reduced or absent, branching still occurred – a contradiction to our earlier findings that luminal pMoesin is necessary for terminal cell morphogenesis[35]. We therefore also tested the effect of abolishing Moesin in terminal cells expressing λBtl. Immunostaining analysis showed that both total Moesin (Fig. 6B′) and pMoesin (Fig. 6D′) were strongly reduced in the *moe*-RNAi and λBtl co-expressing terminal cells. Surprisingly, Moesin depletion also did not suppress the overbranching phenotype caused by λBtl. Therefore, the branching induced by excessive FGF signaling appears not to rely on the same subcellular mechanisms as normal, physiological branching, perhaps because it overrides homeostatic control mechanisms. This is also consistent with the published observation that excessive FGF signaling overrides the requirement for SRF [50]. We therefore cannot use this approach to determine the epistatic relationship between Btl, Moesin and Slik, and it remains open whether Btl acts on Moesin via Slik or through an independent pathway.

### Breathless regulates Moesin independent of the MAP kinase pathway

**Figure 7:**
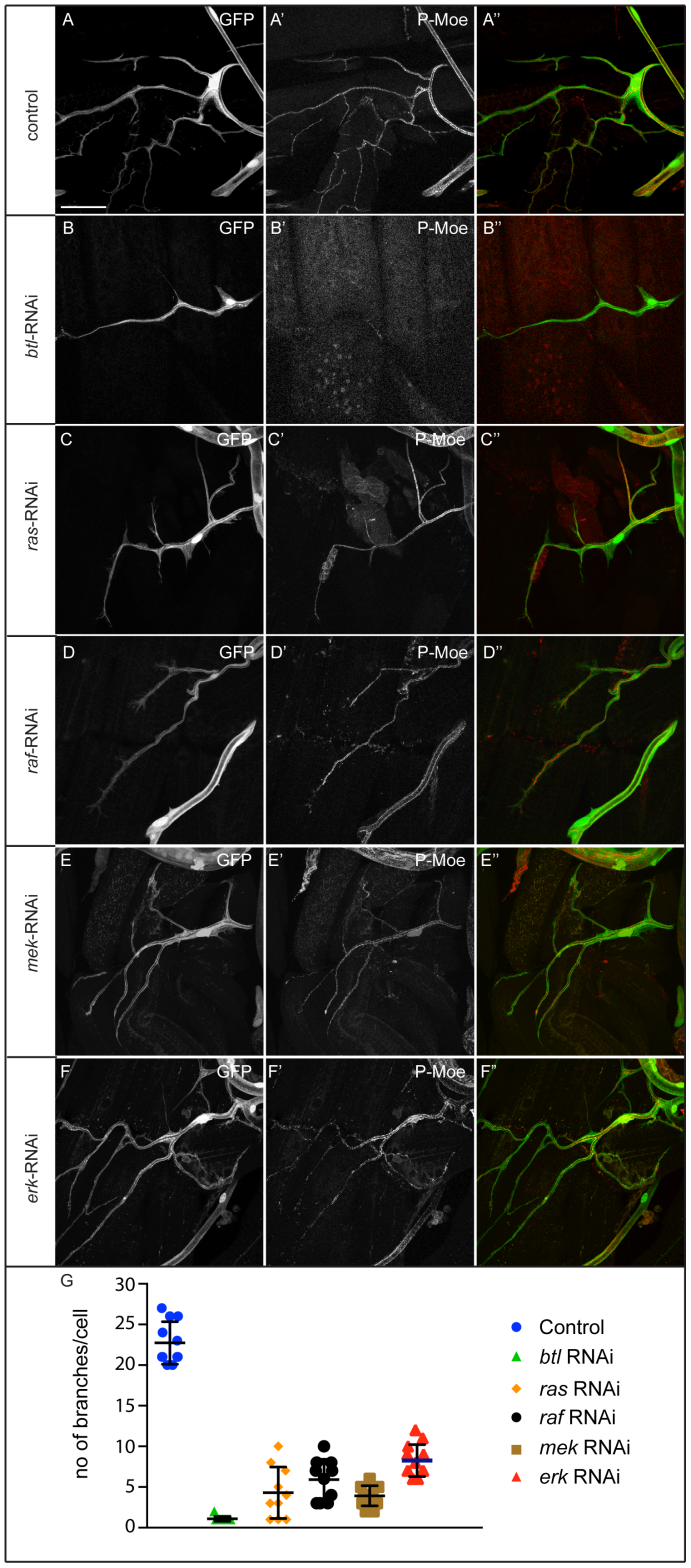
Breathless regulation of pMoesin does not involve the MAPK pathway signalling. (A–F″) pMoesin staining in control, *btl*-RNAi, *ras*-RNAi, *raf*-RNAi, *mek*-RNAi and *erk*-RNAi expressing terminal cells. pMoesin staining is not seen at the luminal membrane in *btl*-RNAi (B′). Depletion of Ras (C′), Raf (D′), Mek (E′) and Erk (F′) does not affect pMoesin levels in terminal cells. (G) depletion of Btl, Ras, Raf, Mek or Erk resulted in reduced numbers of terminal branches. An average of 10 terminal cells were analyzed for each genotype. A–F″ are projections of confocal image stacks. Scale bars: (A–F″) 50 µm.

For a more general test on the role of the MAPK cascade as a potential intermediate in Btl-mediated activation of Moesin, we expressed RNAi constructs targeting the canonical MAPK pathway components Ras, Raf, Mek or Erk. Knockdown of each of the components caused a reduction in the number of terminal cell branches (Fig. 7C–F″ and G) although not as severe as observed in *btl*-RNAi (Fig. 7B–B″ and G), illustrating the important role of the MAPK pathway in terminal cell development. However, the effect on Moesin phosphorylation differed significantly from that caused by *btl*-RNAi. Depletion of the MAPK pathway components Ras, Raf, Mek or Erk did not result in loss of luminal membrane pMoesin as seen in *btl*-RNAi (Fig. 7B′,C′, D′, E′ and F′). Cells with severe branching defects showed proper Moesin phosphorylation and localization (Fig. 7C–F″). To ascertain that the phosphorylation of Moesin was not due to residual MAPK activity caused by incomplete efficiency of the knockdown, we also blocked the pathway using dominant negative constructs of Ras. We tested both *Drosophila* Ras85D^N17^ and mammalian Ras^ N17^. Overexpression of the mammalian construct resulted in first instar larval lethality. Overexpression of *Drosophila* Ras-DN also resulted in lethality, but we managed to obtain some late third instar larvae for analysis when the embryos and early instars were grown at 18°C and only later shifted to 29°C. While the overall morphology of these cells was similar to Btl-RNAi cells, showing that the MAPK pathway is necessary for terminal cell growth, Moesin activation at the luminal membrane was not affected (Fig. S6C′ and D′). These results show that in this context Btl does not act exclusively through the MAPK cascade and that Btl-signaling through the MAPK cascade can be separated from the effect of Btl on the phosphorylation of Moesin.

## Discussion

The cytoskeleton plays important roles in orchestrating the development of different types of biological tubes, including the development of *Drosophila* tracheal branches and tubes. A number of cytoskeleton-organizing molecules have been described as critical regulators of subcellular tube formation, tube stabilization and branching of terminal tracheal cells [35], [63], [76], [79]–[81].

Here we show that activation of Moesin in *Drosophila* tracheal cells is regulated by the serine/threonine kinase Slik. In the terminal tracheal cells, Slik is required for spatially restricted activation of Moesin at the luminal membrane and this interaction between Slik and Moesin is crucial for normal branching and tube formation. This function of Slik is consistent with earlier reports on the role of Slik in the activation of Moesin in other cells and tissues [43]–[45]. While the Slik-Moesin interaction is important for terminal cell development, we do not rule out the possibility of the requirement for other substrates of Slik. Merlin, one other known substrate of Slik, is expressed in tracheal cells and its depletion causes defects, but it is not clear whether Merlin acts in the same pathway as Slik and Moesin. The possible role of Merlin in terminal cell development will need to be characterized particularly in the context of Merlin's function in signaling receptor endocytosis at the plasma membrane and its interaction with the Hippo signaling pathway [82]–[85].

ERM proteins have a conserved threonine residue (T559) in their C-Terminal FERM domain, a target of Slik kinase in *Drosophila* Moesin [44], [45], and phosphorylation of this conserved threonine residue is critical for ERM protein activation and function. There are examples of additional phosphorylation targets necessary for ERM protein function and dispensability of the conserved threonine residue in specific contexts [22], [46]. Compromising Slik function in terminal cells abrogated activation of Moesin at the luminal membrane and resulted in a reduction of branches. However, Slik depletion did not lead to the complete failure of branching as seen in the case of Moesin depletion, suggesting other Moesin-activating molecules or pathways operating in terminal cells. We find that in addition to Slik, the FGF receptor Breathless is required for spatially restricted activation of Moesin at the luminal membrane in the terminal cells.

During embryonic tracheal development, the actin-binding protein Singed, the *Drosophila* homologue of Fascin, regulates branch migration, terminal branching and branch fusion. This involves actin organization in filopodia at the migrating fronts of the embryonic terminal cells [63]. Singed is also transcriptionally upregulated by Btl in terminal cells [63]. In the *btl*-RNAi knockdown experiments we found that total Moesin was not reduced in the Btl-depleted terminal cells, suggesting that the effect of Btl on Moesin was not at the transcriptional level.

It is also unlikely that Btl directly or indirectly affects the luminal membrane localization of non-activated Moesin, for example by acting as a membrane anchor, since Moesin remains enriched at the luminal membrane in Btl-depleted cells. Finally, the unchanged levels of Slik in Btl-depleted cells indicate that Btl does not regulate Moesin activation indirectly through transcriptional regulation of Slik. With the possibilities of transcriptional regulation of Moesin and Slik ruled out, Btl most likely acts by direct or indirect post-transcriptional regulation of Moesin in the terminal cells. It could phosphorylate Moesin on a tyrosine in an activation event upstream of the Slik dependent threonine-phosphorylation, it might activate Slik, or it might act through other intermediates. While threonine-phosphorylation is important for ERM activation and function, in some cases threonine-phosphorylation is dispensable and alternate ERM-activating mechanisms are used [22], [46]. Whether Btl-mediated activation of Moesin terminal cells involves a direct or an indirect interaction and if it includes activation of additional amino acids upstream of the conserved threonine residue are interesting open questions to be addressed in the future.

## Supporting Information

Figure S1 (A and B) Larval fillet preparations from control (A) and *slik* ^1^ homozygous larvae (B). (C–D″) Merlin immunostaining in control terminal cell (C′) in third instar larvae. (D–D″) Single focal plane from an image stack showing Merlin staining (D′) in the terminal branches. Merlin is distributed throughout the terminal cell (C′ and D′). Merlin-depleted terminal cells have larger masses of cytoplasm around the nucleus (F) than control cells (E). (C–C″, E and F) are projections of confocal image stacks. Scale bars: (C–C″, E, F) 30 µm, (D–D″) 5 µm.
(TIF)

Figure S2 **Distribution of Merlin in tracheal cells.** Control (A,B) and Slik-depleted (C,D) terminal cells stained with antibodies against Merlin. Merlin is seen throughout the cell with enrichments in a punctate pattern both in control and in Slik-depleted cells. It is also detected in the muscles surrounding the tracheal cells. (B) and (D) are higher magnifications of details from (A) and (C). Scale bar: (A–A″ and C–C″) 50 µm, (B–B″ and D–D″) 20 µm.
(TIF)

Figure S3 **Two examples of Btl-depleted terminal cells with long cytoplasmic extensions without any visible lumen.** The white arrowheads mark the point where the visible lumen ends. Scale bar: 30 µm.
(TIF)

Figure S4 (A–B″) Examples of pMoesin staining in Btl-depleted terminal cells. In 63% of Btl-depleted cells luminal membrane localized pMoesin is absent (A′) or reduced (B′ and B″). (B) shows that even two cells in the same larva can exhibit different levels of pMoesin, even though they are of similar size and have similar lumens. pMoesin is also seen in other tissues surrounding the tracheal cells. Scale bar: (A–A″) 50 µm, (B–B″) 30 µm.
(TIF)

Figure S5 **Effect of FGF signaling on the luminal membrane of terminal cells.** (A, B) Control (A) and Btl-depleted (B) cells stained with antibodies against the luminal membrane protein Pio. The localization of Pio to the luminal membrane is not affected when Btl signaling is disrupted. (C,D) Knockdown of Slik in the presence of over-activation of FGF signaling. Tracheal cells expressing the constitutively active FGF receptor λ*btl* together with *slik*-RNAi. Pio is properly localized at the luminal membrane despite compromised Slik signaling. D shows a higher magnification of the area marked with the white box. Scale bar: (A–C″) 50 µm, (D–D″) 20 µm.
(TIF)

Figure S6 **Effect of disrupting Ras signaling in terminal cells.** Tracheal cells from control larvae (A) or larva tracheal cells expressing Btl-RNAi (B) or dominant negative Ras (C,D) stained for pMoesin. Depletion of Btl and expression of RasDN lead to branching defects. While pMoesin is not detected in Btl-depleted cells, it is seen in its normal location at the luminal membrane in cells express RasDN. C and D show examples at the extremes of the range of phenotypes: some cells develop not branches at all, while some develop a small number of short branches. Scale bar: (A–D″) 50 µm.
(TIF)
