# Supplementary figures and images for: Slik and the Receptor Tyrosine Kinase Breathless Mediate Localized Activation of Moesin in Terminal Tracheal Cells
Source: PLoS One. 2014 Jul 25;9(7):e103323. doi: 10.1371/journal.pone.0103323 (PMC4111555; doi:10.1371/journal.pone.0103323)

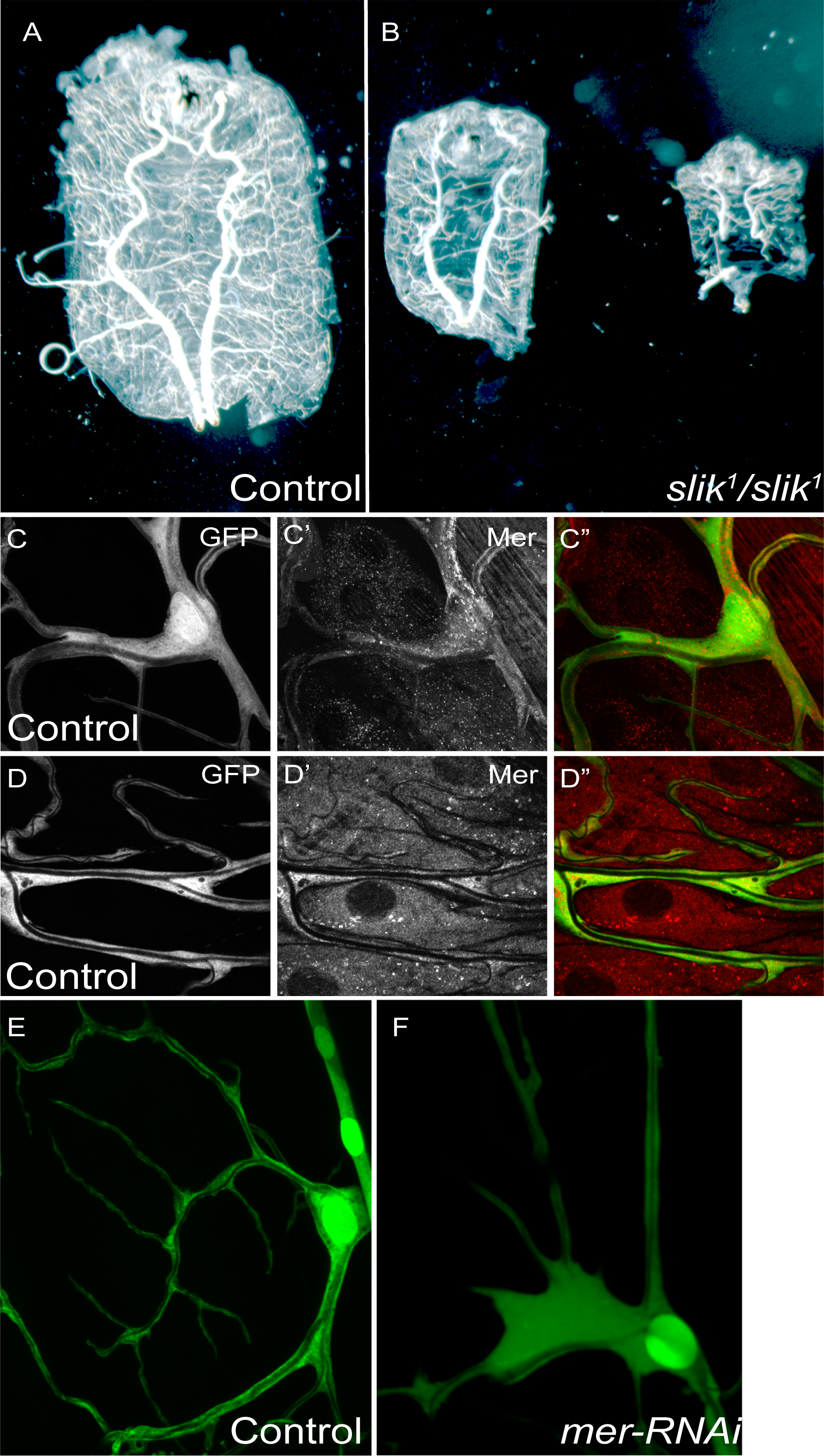

Supplement: Figure S1 — (A and B) Larval fillet preparations from control (A) and slik 1 homozygous larvae (B). (C–D″) Merlin immunostaining in control terminal cell (C′) in third instar larvae. (D–D″) Single focal plane from an image stack showing Merlin staining (D′) in the terminal branches. Merlin is distributed throughout the terminal cell (C′ and D′). Merlin-depleted terminal cells have larger masses of cytoplasm around the nucleus (F) than control cells (E). (C–C″, E and F) are projections of confocal image stacks. Scale bars: (C–C″, E, F) 30 µm, (D–D″) 5 µm. (TIF) [file pone.0103323.s001.tif]

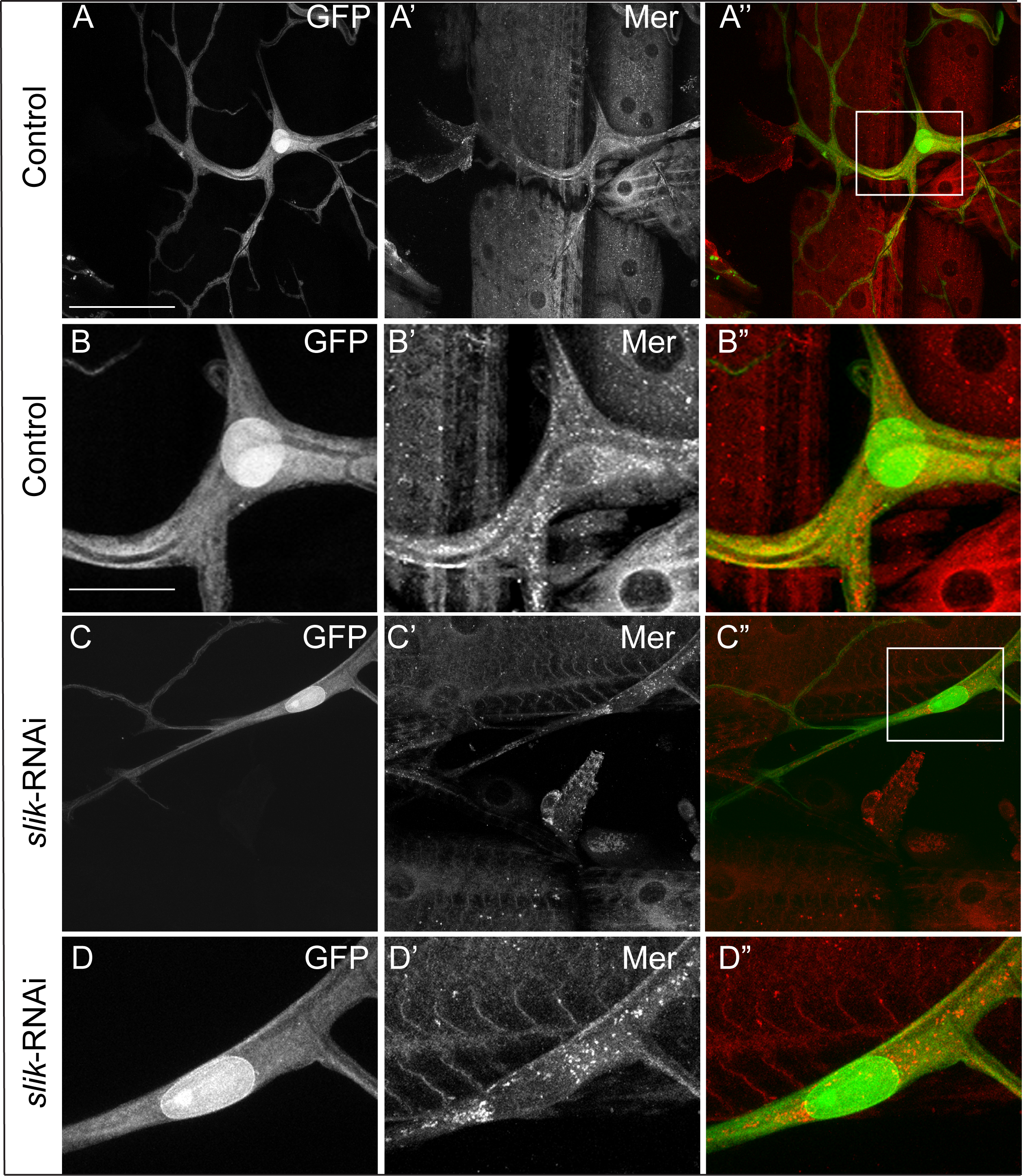

Supplement: Figure S2 — Distribution of Merlin in tracheal cells. Control (A,B) and Slik-depleted (C,D) terminal cells stained with antibodies against Merlin. Merlin is seen throughout the cell with enrichments in a punctate pattern both in control and in Slik-depleted cells. It is also detected in the muscles surrounding the tracheal cells. (B) and (D) are higher magnifications of details from (A) and (C). Scale bar: (A–A″ and C–C″) 50 µm, (B–B″ and D–D″) 20 µm. (TIF) [file pone.0103323.s002.tif]

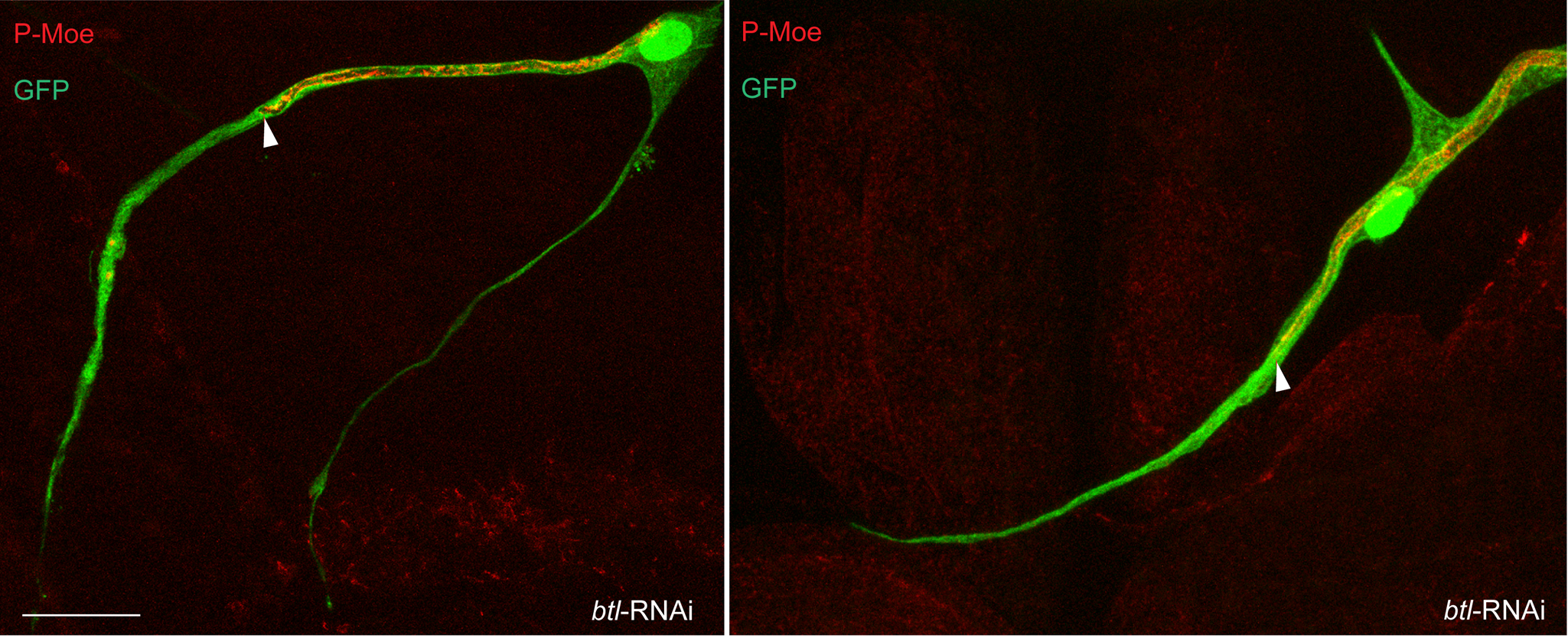

Supplement: Figure S3 — Two examples of Btl-depleted terminal cells with long cytoplasmic extensions without any visible lumen. The white arrowheads mark the point where the visible lumen ends. Scale bar: 30 µm. (TIF) [file pone.0103323.s003.tif]

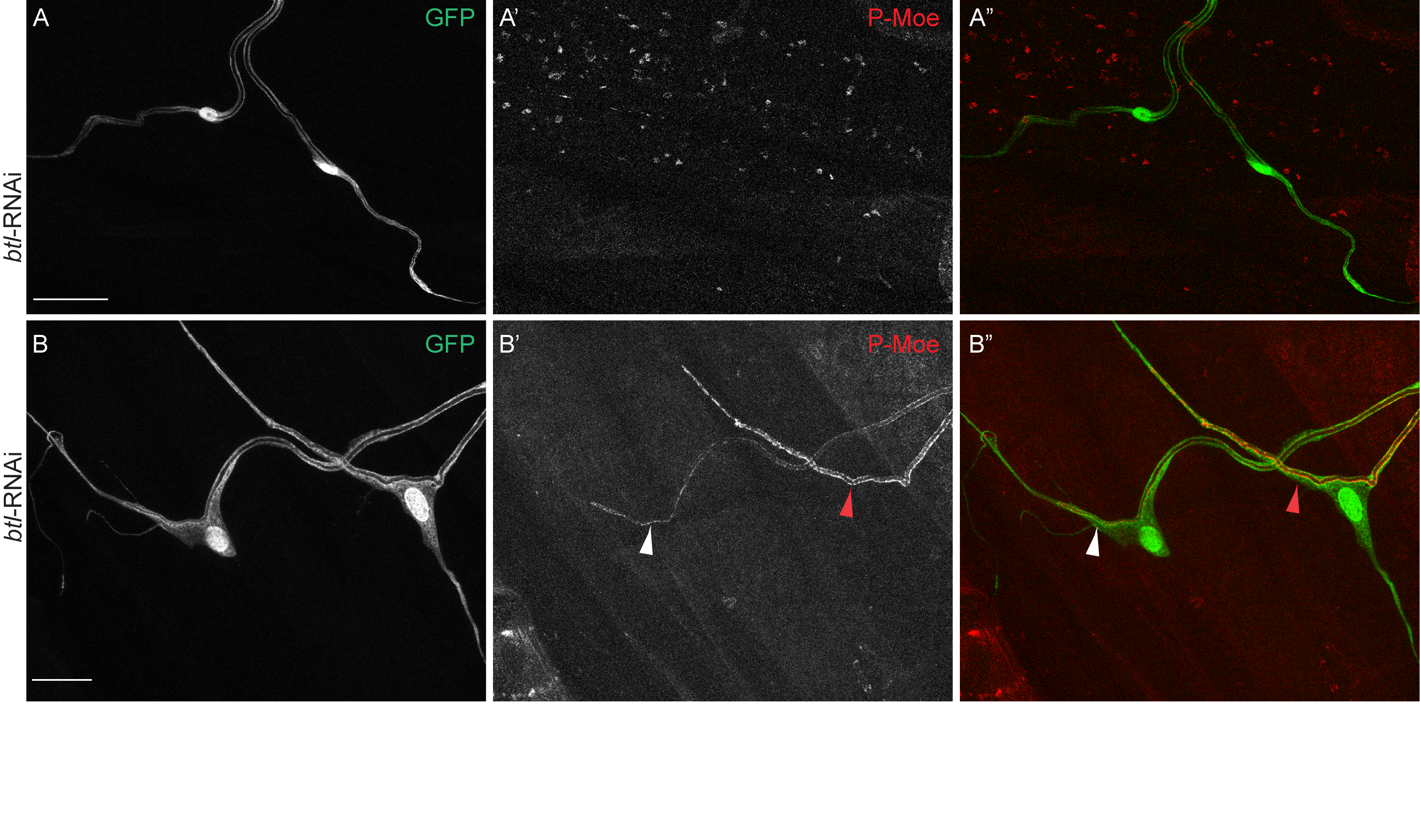

Supplement: Figure S4 — (A–B″) Examples of pMoesin staining in Btl-depleted terminal cells. In 63% of Btl-depleted cells luminal membrane localized pMoesin is absent (A′) or reduced (B′ and B″). (B) shows that even two cells in the same larva can exhibit different levels of pMoesin, even though they are of similar size and have similar lumens. pMoesin is also seen in other tissues surrounding the tracheal cells. Scale bar: (A–A″) 50 µm, (B–B″) 30 µm. (TIF) [file pone.0103323.s004.tif]

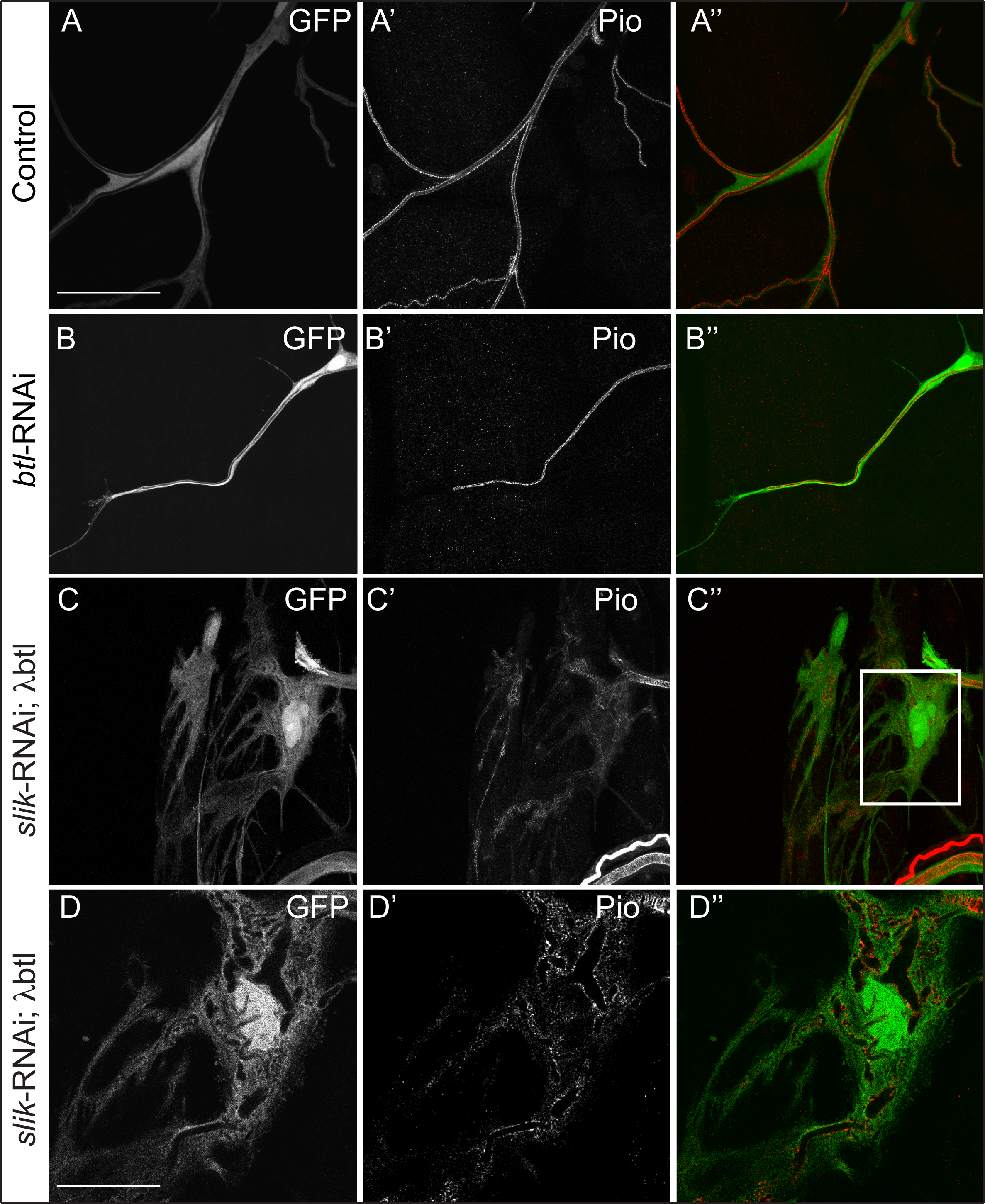

Supplement: Figure S5 — Effect of FGF signaling on the luminal membrane of terminal cells. (A, B) Control (A) and Btl-depleted (B) cells stained with antibodies against the luminal membrane protein Pio. The localization of Pio to the luminal membrane is not affected when Btl signaling is disrupted. (C,D) Knockdown of Slik in the presence of over-activation of FGF signaling. Tracheal cells expressing the constitutively active FGF receptor λbtl together with slik-RNAi. Pio is properly localized at the luminal membrane despite compromised Slik signaling. D shows a higher magnification of the area marked with the white box. Scale bar: (A–C″) 50 µm, (D–D″) 20 µm. (TIF) [file pone.0103323.s005.tif]

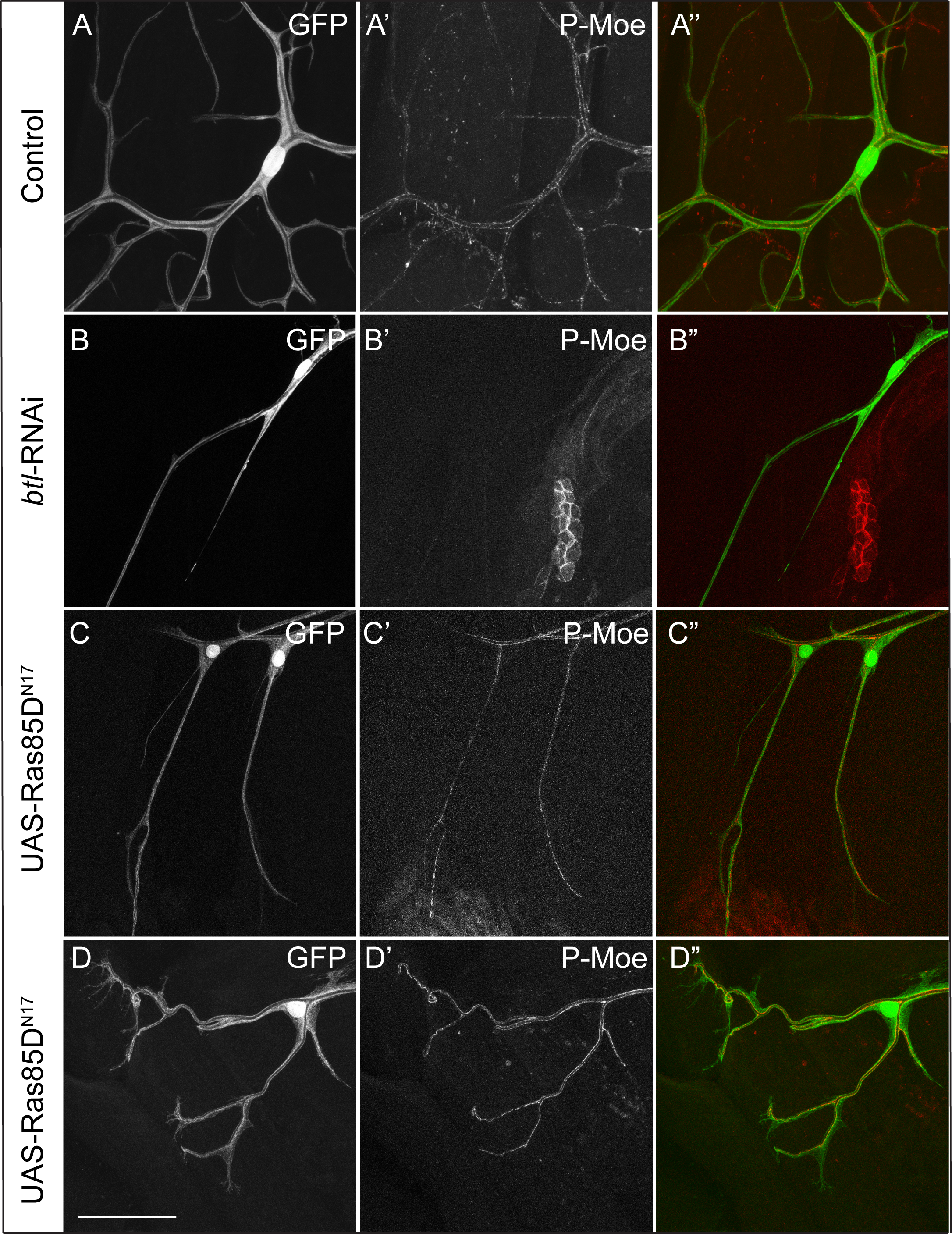

Supplement: Figure S6 — Effect of disrupting Ras signaling in terminal cells. Tracheal cells from control larvae (A) or larva tracheal cells expressing Btl-RNAi (B) or dominant negative Ras (C,D) stained for pMoesin. Depletion of Btl and expression of RasDN lead to branching defects. While pMoesin is not detected in Btl-depleted cells, it is seen in its normal location at the luminal membrane in cells express RasDN. C and D show examples at the extremes of the range of phenotypes: some cells develop not branches at all, while some develop a small number of short branches. Scale bar: (A–D″) 50 µm. (TIF) [file pone.0103323.s006.tif]
